# Ultrasound in the Emergency Department Identifies Ectopic Pregnancy Post Hysterectomy: A Case Report

**DOI:** 10.5811/cpcem.2022.2.54929

**Published:** 2022-05-05

**Authors:** Allison Cohen, Dorothy Shi, Evan Keraney, Brendon Stankard, Mathew Nelson

**Affiliations:** North Shore University Hospital, Department of Emergency Medicine, Manhasset, New York

**Keywords:** point-of-care ultrasound, ectopic pregnancy, case report

## Abstract

**Introduction:**

Ruptured ectopic pregnancy is one of the leading causes of maternal death. Point-of-care ultrasound (POCUS) has been shown to be highly sensitive for excluding ectopic pregnancy. Ectopic pregnancy after a hysterectomy is a rare but life-threatening occurrence. We present a case where POCUS helped to diagnose a post-hysterectomy ectopic pregnancy.

**Case report:**

A 36-year-old female with a prior surgical history of hysterectomy without oophorectomy presented to the emergency department with lower abdominal pain. A POCUS revealed free fluid in the right upper quadrant with an unremarkable gallbladder. Subsequently, the pelvic POCUS noted free fluid as well as a heterogeneous structure in the right adnexa. The clinician ordered a serum beta human chorionic gonadotropin level, which was 173.2 international units per milliliter (lU/mL) (negative: < 5m IU/ml). Transvaginal ultrasound revealed a right adnexal echogenic structure with surrounding vascularity and moderate, complex free fluid suggestive of hemorrhage. Given the concern for possible ectopic pregnancy, obstetrics took the patient to the operating room where a right tubal ectopic pregnancy was confirmed.

**Conclusion:**

A ruptured ectopic pregnancy is a life-threatening condition that requires rapid diagnosis. Ectopic pregnancy post hysterectomy is an uncommon occurrence infrequently considered in the differential diagnosis of lower abdominal pain, leading to considerable delays in diagnosis. Although uncommon, emergency clinicians must consider this diagnosis in female patients with lower abdominal pain.

## INTRODUCTION

Ectopic pregnancy occurring after a hysterectomy is a rare occurrence that is often difficult to diagnose and is associated with high morbidity and mortality. This diagnosis is often challenging as presenting symptoms are non-specific. There have been multiple reported cases in the literature of ectopic pregnancies occurring post hysterectomy,^1^,^4^,^8^,^16^ with the majority occurring during the time interval directly following the procedure.^1^ This case demonstrates how point-of-care ultrasound (POCUS) helped to diagnose a patient with a post-hysterectomy ectopic pregnancy.

## CASE REPORT

**Image 1:**
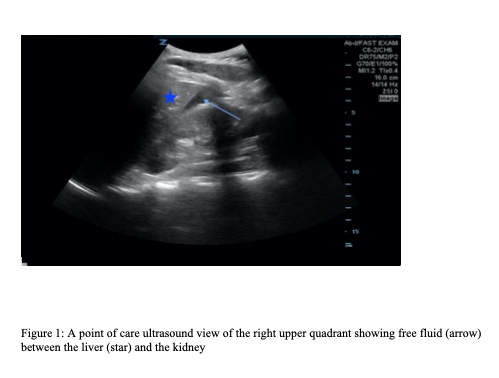
A point-of-care ultrasound view of the right upper quadrant showing free fluid (arrow) between the liver (star) and kidney.

A 36-year-old gravida 4 para 2-0-2-2 female, who had a history of ovarian cysts and cervical cancer with prior total laparoscopic hysterectomy without oophorectomy three months prior, presented to the emergency department (ED) with a chief complaint of lower abdominal pain. The patient reported severe lower abdominal pain that started earlier that day after intercourse. The pain radiated to the right upper quadrant and was worse with deep inspiration. The pain was associated with nausea and vomiting. The patient denied any fevers, chills, chest pain, shortness of breath, dysuria, vaginal bleeding, or vaginal discharge. She had taken acetaminophen prior to ED arrival without any improvement. On presentation the patient was hemodynamically stable with a blood pressure of 129/85 millimeters of mercury, a heart rate of 76 beats per minute, and temperature of 36.7° Celsius. Physical exam was significant for lower abdominal and right upper quadrant tenderness. A point-of-care ultrasound (POCUS) of the right upper quadrant was performed initially, which revealed free fluid (Image 1) and an unremarkable gallbladder.

**Image 2:**
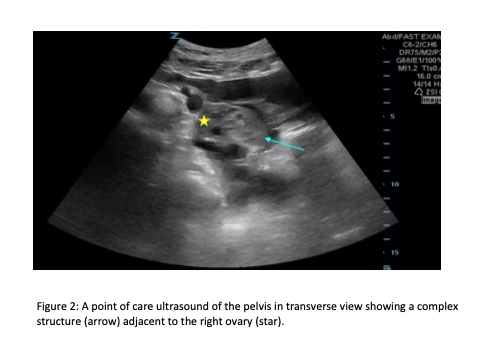
Point-of-care ultrasound of the pelvis in transverse view showing a complex structure (arrow) adjacent to the right ovary (star).

Given the presence of free fluid in the upper abdomen, and because the patient was a female of childbearing age who reported lower abdominal pain as well, the sonographer expanded the exam to evaluate the lower abdomen. In the right lower abdomen, a heterogeneous structure was visualized in the right adnexa surrounded by complex free fluid (Image 2). Transvaginal POCUS was then performed showing a right adnexal echogenic structure with surrounding vascularity and complex free fluid concerning for a ruptured ectopic pregnancy. A beta human chorionic gonadotropin was ordered after performing the ultrasound given the high clinical suspicion for an ectopic pregnancy.

**CPC-EM Capsule**

What do we already know about this clinical entity?

*Ectopic pregnancy post hysterectomy is a rare but life-threatening occurrence. The majority occur directly following the procedure*.

What makes this presentation of disease reportable?

*Post-hysterectomy ectopic pregnancy is difficult to diagnose and is associated with morbidity. Point-of-care ultrasound (POCUS) helped to make this diagnosis*.

What is the major learning point?

*Erroneously, ectopic pregnancy is often removed from the differential diagnosis in patients who are post hysterectomy, leading to a significant delay in diagnosis*.

How might this improve emergency medicine practice?

*Increased mortality rates in patients with late post-hysterectomy ectopic pregnancy make rapid detection with POCUS essential to expedite treatment*.

Laboratory values showed no evidence of anemia with a hemoglobin of 12.8 grams per deciliter (g/dL) (reference range: 11.5–15.5 g/dL) and a mild leukocytosis of 12.4 kilo per microliter (K/uL) (3.8–10.5 K/uL). The human gonadotropin level returned at 173.2 milli-international units per milliliter (mlU/mL) (negative: < 5 mIU/mL). Obstetrics was consulted and took the patient to the operating room. The patient was found to have a ruptured right fallopian-tube ectopic pregnancy with approximately 200 mL of intraperitoneal blood. In the operating room she received bilateral salpingectomy with removal of the ectopic pregnancy. The patient had an uneventful recovery after the procedure and was discharged home the next day without further complications.

## DISCUSSION

Ectopic pregnancy after a hysterectomy is a rare but life-threatening condition that requires rapid diagnosis. Erroneously, ectopic pregnancy is often removed from the differential diagnosis in patients who are post hysterectomy, leading to a significant delay in making this critical diagnosis. The presenting symptoms of post-hysterectomy ectopic pregnancy are generally non-specific and range from lower abdominal pain to nausea and vomiting. Vaginal bleeding, which is a common presenting symptom of ectopic pregnancy in a patient with a uterus, is relatively uncommon in post-hysterectomy ectopic pregnancies and it was the presenting symptom in only a few reported cases.^2^

While post-hysterectomy ectopic pregnancies are rare, there have been reported cases leading to a further classification of the phenomenon as either early or late.^8^,^17^,^18^ Early presentations occur when either conception or potential for conception occurs at the time of the hysterectomy.^2^,^3^ In these cases, fertilization occurred prior to the hysterectomy. It has previously been reported that many of these patients who present soon after the hysterectomy procedure are often diagnosed with postoperative complications leading to a delay in the diagnosis of ectopic pregnancy.^4^

Late presentation ectopic pregnancy occurs well after the hysterectomy has been completed and can occur up to 12 years post procedure.^2^,^4^ Although the pathophysiology remains unclear, cmultiple theories have been proposed. One potential mechanism is the creation of a fistula tract between the vaginal dome and the peritoneum or vaginal dome and the fallopian tube.^5^,^6^ This would create a passageway for sperm to fertilize an ovum. Another possible mechanism is that the fallopian tube prolapses into the vagina creating a communication between the two structures and allowing for fertilization to occur. Although the majority of documented post-hysterectomy ectopic pregnancies occurred after vaginal hysterectomy, it can still be diagnosed in patients after an abdominal hysterectomy with the most common location being in the fallopian tube 7,8

Early ordering of beta human chorionic gonadotropin levels can aid emergency physicians (EP) in making this diagnosis. In the majority of previously reported post-hysterectomy ectopic pregnancy cases the diagnosis was often delayed and unexpected.^4^ An additional tool that can aid EPs in making this diagnosis is early use of POCUS. In the ED, POCUS is a useful diagnostic tool for evaluating undifferentiated causes of abdominal pain. Point-of-care ultrasound has been shown to be helpful in confirming intrauterine pregnancy and recognizing hemorrhage due to an ectopic pregnancy. Prior studies have shown that EPs can accurately and rapidly use POCUS to detect an intrauterine pregnancy, and previous data has shown a clear role for early ultrasound in patients at risk for ectopic pregnancy.^9^,^10^,^11^,^12^

Early diagnosis of a ruptured ectopic pregnancy is essential as it is a leading cause of first-trimester maternal death. Additionally, there have been reports of increased mortality rates in patients with late post-hysterectomy ectopic pregnancy compared with those with intact uteri ectopic pregnancy.^8^,^13^ Therefore, rapid and accurate detection is essential to reduce mortality and expedite treatment.^7^

## CONCLUSION

Ectopic pregnancies in patients with a previous hysterectomy are rare occurrences making the diagnosis difficult. Although it is often on the differential for female patients presenting with abdominal pain, it may be overlooked in patients with a history of a hysterectomy. This case demonstrates the use of point-of-care ultrasound in helping to diagnose a ruptured ectopic pregnancy in a patient with a prior hysterectomy. In this case the findings seen on the POCUS exam facilitated early consultation, resuscitation, and operative intervention. Additionally, as the rate of hysterectomy in child-bearing women is rising, it is expected that the incidence of ectopic pregnancy post hysterectomy will increase.^8^ It is, therefore, important for emergency physicians to remember that prior hysterectomy does not exclude the diagnosis of ectopic pregnancy, and thus EPs must continue to have a high clinical suspicion for this pathology.
